# Correlation-based network analysis combined with machine learning techniques highlight the role of the GABA shunt in *Brachypodium sylvaticum* freezing tolerance

**DOI:** 10.1038/s41598-020-61081-4

**Published:** 2020-03-11

**Authors:** David Toubiana, Nir Sade, Lifeng Liu, Maria del Mar Rubio Wilhelmi, Yariv Brotman, Urszula Luzarowska, John P. Vogel, Eduardo Blumwald

**Affiliations:** 1 Department of Plant Sciences, University of California, Davis, California USA; 2 School of Plant Sciences and Food Security, Tel Aviv University, Tel Aviv, Israel; 3 DOE Joint Genome Institute, 2800 Mitchell Dr., Walnut Creek, CA 94598 USA; 4 Department of Life Sciences, Ben Gurion University of the Negev, Beer Sheva, Israel

**Keywords:** Biological techniques, Molecular biology, Plant sciences, Systems biology

## Abstract

Perennial grasses will account for approximately 16 billion gallons of renewable fuels by the year 2022, contributing significantly to carbon and nitrogen sequestration. However, perennial grasses productivity can be limited by severe freezing conditions in some geographical areas, although these risks could decrease with the advance of climate warming, the possibility of unpredictable early cold events cannot be discarded. We conducted a study on the model perennial grass *Brachypodium sylvaticum* to investigate the molecular mechanisms that contribute to cold and freezing adaption. The study was performed on two different *B. sylvaticum* accessions, Ain1 and Osl1, typical to warm and cold climates, respectively. Both accessions were grown under controlled conditions with subsequent cold acclimation followed by freezing stress. For each treatment a set of morphological parameters, transcription, metabolite, and lipid profiles were measured. State-of-the-art algorithms were employed to analyze cross-component relationships. Phenotypic analysis revealed higher adaption of Osl1 to freezing stress. Our analysis highlighted the differential regulation of the TCA cycle and the GABA shunt between Ain1 and Osl1. Osl1 adapted to freezing stress by repressing the GABA shunt activity, avoiding the detrimental reduction in fatty acid biosynthesis and the concomitant detrimental effects on membrane integrity.

## Introduction

The increase in energy demand coupled with the environmental impact of fossil fuels has led to the expansion of programs using perennial species as an alternative source of energy. In this context, perennial grasses, traditionally used as forage and turf, have become the preferred candidates for biomass cropping systems attributed to their ability to grow under low input conditions and high interception of sunlight^1^. Predictions showed that perennial grasses will account for approximately 16 billion gallons of renewable fuels by the year 2022^2,3^. Perennial grasses have shown to support carbon (C) and nitrogen (N) sequestration in the soil^4–6^, contributing to mitigate climate change and environmental degradation^7–9^. *Brachypodium sylvaticum* is a perennial grass with a simple genome, self-fertility, a short life cycle, and low growth requirements. Its recently released genome, expression gene atlas^10^ and well established genetic transformation have made it an efficient model to study the genetic, molecular, and physiological components of perennial grasses^11,12^.

Unfavorable winter conditions, including freezing, limit the geographical range of many important crops and reduce their productivity due to growth repression^13,14^. Low temperatures combined with high light intensity provoke the inhibition of the photosystem II and ROS production leading to oxidative stress^15^. In addition, ice formation in the apoplast induces both, cell membrane integrity stress and osmotic stress^16^. The plant’s response to low temperatures involves extensive reprograming of gene expression via transcription factors^17^, resulting in concerted shifts of the metabolic network. Moreover, plants respond to cold stress by increasing the degree of unsaturation of the membrane lipids and by remodeling lipids, which in turn may help the plant to avoid membrane integrity damage and achieve chilling tolerance^18^. The molecular and biochemical pathways regulating winter-hardiness in perennial grasses are poorly understood and deserve further attention.

With the advent of high-throughput platforms in the biological sciences, data driven analysis has become a key element for the integration of biological data of different backgrounds. A plethora of tools have been developed which tackle the complex problem of data integration from different perspectives, e.g.^19–24^. The biological interpretation of the data, however, is always study specific. Many of these tools make use of graph theory and network analysis, in one way or another, as the integration of heterogenic data is accomplished with ease and graph theory is readily equipped with a myriad of analytical and interpretational tools^25–27^. Correlation-based network analysis (CNA) is based on mathematically defined (dis)similarity measures that correlate different components to each other and the resulting correlation coefficients reflect the magnitude of the co-linear relationship of the components. In complex biological data, with thousands of different components assessed at various data points, the correlation is synonymous with coordinated behavior. With the exploration of the structural properties of graphs, CNA has been successfully used to postulate biological hypotheses which have been verified *a posteriori*^28^. Recently, a novel approach that combines CNA with machine learning (ML) techniques, was used to predict the presence of metabolic pathways in tomato correlation networks (CN)^29^.

**Figure 1:**
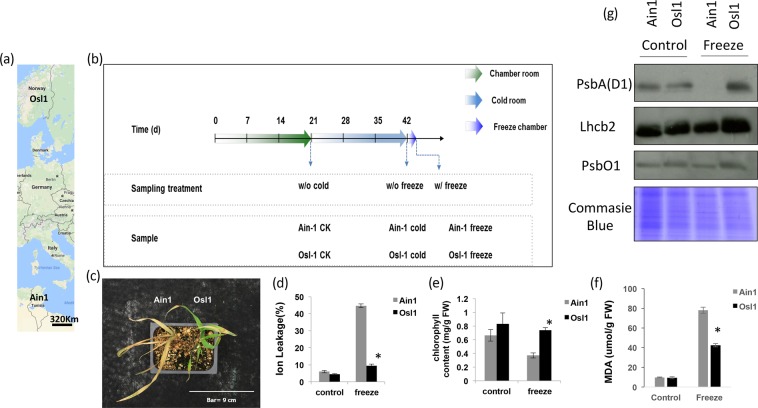
Effect of freezing on two B. Sylvaticum accessions (Ain1 and Osl1). (**a**) Geographical habitat of the B. Sylvaticum accessions (**b**) Experimental setup (**c**) Ain1 and Osl1 phenotype post-stress (**d**) Ain1 and Osl1 Ion leakage and (**e**) Chlorophyll content (**f**) Lipid Peroxidation (MDA) and (**g**) Immunoblot analysis of PSII subunits Lhcb2, PsbA (D1), and PsbO1. Values are Mean ± SE (n = 3–12). The data was analyzed using Student’s t test. Asterisks indicate significant differences (p ≤ 0.05).

Here, we investigated the response of two *Brachypodium sylvaticum* accessions (Osl1 and Ain1) to cold and freezing stress. Accession Osl1 is endemic to Northern Europe, while Ain1 can be predominantly found in Northern Africa (Fig. 1a). We studied traits of the two accessions known to exert different phenotypes. Furthermore, we applied a top-down based approach integrating gene expression data and metabolic and lipid profiles from high throughput platforms and state-of-the-art algorithms for the analysis of big biological data. We employed the aforementioned ‘CNA combined with machine learning techniques’ approach for a comparative analysis between two correlation networks to identify genotype-specific ‘active’ metabolic pathways. Furthermore, we modified a recently developed genetic search algorithm^30^ to integrate gene expression data with metabolic profiles and detect candidate genes that may be key regulators for the identified ‘active’ metabolic pathways.

## Results

### Phenotypic analysis reveals Osl1 freezing tolerance

To morphologically characterize the effect of freezing stress on *B. sylvaticum*, freezing experiments were performed on two different *B. sylvaticum* accessions, Osl1 and Ain1 (Fig. 1a). Plants of both accessions were grown under controlled conditions for 21 days before being cold acclimated for additional 21 days. Subsequently, plants were moved into a freezing chamber for a freezing challenge (Fig. 1b). Seedling leaf samples were taken (i) before cold acclimation, (ii) at cold acclimation, and (iii) during the freezing challenge (Fig. 1b). After the freezing challenge, Ain1 plants were severely damaged showing leaf necrosis while Osl1 remained viable (Fig. 1c). Freezing treatment led to increased ion leakage in both accessions as compared to the non-stressed control samples. However, the increase was more significant for Ain1 as compared to Osl1 (Fig. 1d). Chlorophyll content significantly decreased in Ain1 in response to the freezing treatment, while Osl1 kept higher chlorophyll content compared to Ain1 (Fig. 1e). To test for lipid peroxidation, MDA content was measured. The analysis showed that Ain1 plants displayed higher lipid peroxidation than Osl1 plants (Fig. 1f). Upon exposure to freezing conditions, the degradation of PSII subunits (D1, PsbO1, and Lhcb2) was more evident in Ain1 plants as compared to Osl1 plants (Fig. 1g). Taken together, our results showed that Osl1 had a higher freezing-tolerance than Ain1 accession.

### Differential gene expression analysis highlights freezing tolerance of Osl1

**Figure 2:**
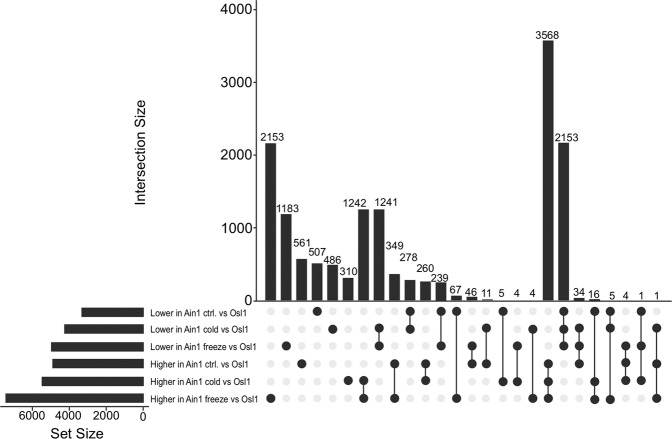
Intersections among stress differentially expressed genes across accessions and treatments. Number above bars indicates the number of genes within each intersection. Set size indicates the total number of significant genes in the treatments.

To gain an insight into differential gene expression profiles between the two accessions and their respective treatments, cDNA libraries were prepared and sequenced via a Illumina Hiseq platform, followed by differential gene expression analysis^31^. In total, 14,727 differentially expressed genes (DEGs) were identified when comparing the different treatments of Ain1 and Osl1 samples (Fig. 2 - for a full list of genes IDs see Supplementary Data S1). The comparison of Ain1 control vs. Osl1 control samples identified 561 up-regulated DEGs and 517 down-regulated DEGs. The comparison between Ain1 cold vs. Osl1 cold samples identified 310 up-regulated DEGs and 486 down-regulated DEGs. The comparison between Ain1 vs. Osl1 samples during freezing identified 2,153 up-regulated DEGs and 1,183 down-regulated DEGs.

In summary, the differential gene expression analysis demonstrated that the majority of up-regulated DEGs were specific to Ain1 samples as compared to Osl1 samples, i.e. among the 5,721 unique DEGs resulting from the comparison between Ain1 and Osl1 treatments, 3,568 were up-regulated in Ain1 while 2,153 were up-regulated in Osl1. In particular, the freezing treatment exerted the greatest impact on gene expression in Ain1 supporting the notion of cold adaptation of the Osl1 ecotype.

### Metabolite and lipid profiles are treatment specific

**Figure 3:**
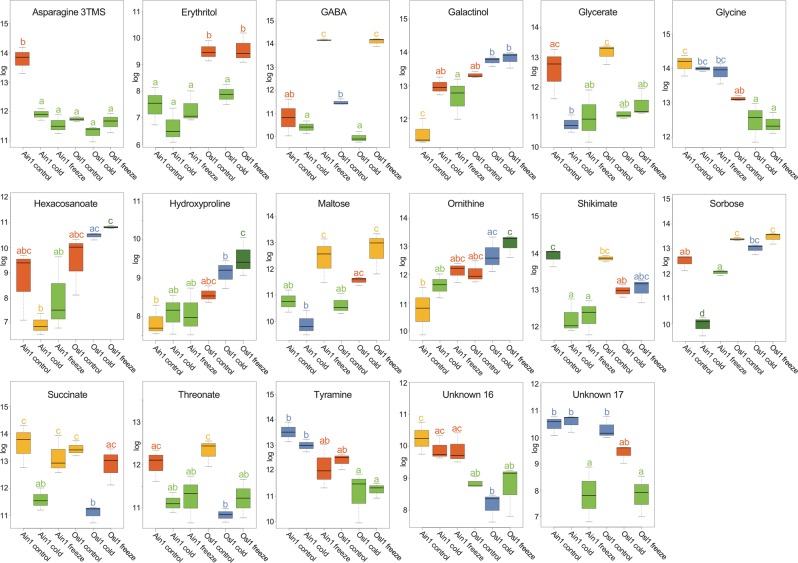
Significantly changing metabolites. Illustrated are the levels of the 17 significantly changing metabolites identified via analysis of variance in two *Brachypodium sylvaticum* accessions across different temperature treatments. X-axes represent the log of relative content, y-axes represent different accessions and their respective treatments. Values are the log(mean) ± SE (n = 3). Different letters and color codes indicate significant differences (p ≤ 0.05) according to posthoc Tukey’s test.

In an effort to assess metabolic changes between the accessions and their respective treatments, metabolite profiles were generated using a GC-MS platform as well as lipid profiles using an LC-MS platform. In total, 139 metabolites (Supplementary Fig. 1) and 195 lipids (Supplementary Fig. 2) were unequivocally identified. One-factorial analysis of variance revealed significant differences for 17 metabolites (Fig. 3) and 53 lipids (Supplementary Fig. 3a–c); i.e. two out of six diacylglycerols, six out of 22 digalactosyl-diacylglycerols, eight of 19 monogalactosyldiacylglycerols, five out of 28 phosphatidylcholines, four out of 15 phosphatidylethanolamines, eight out of 21 phosphatidylglycerols, one out of five^32^ phosphatidylinositols, four out of 15 phosphatidylserines, six out of 12 sulfoquinovosyldiacylglycerols, and six out of 52 triacylglycerides.

**Figure 4:**
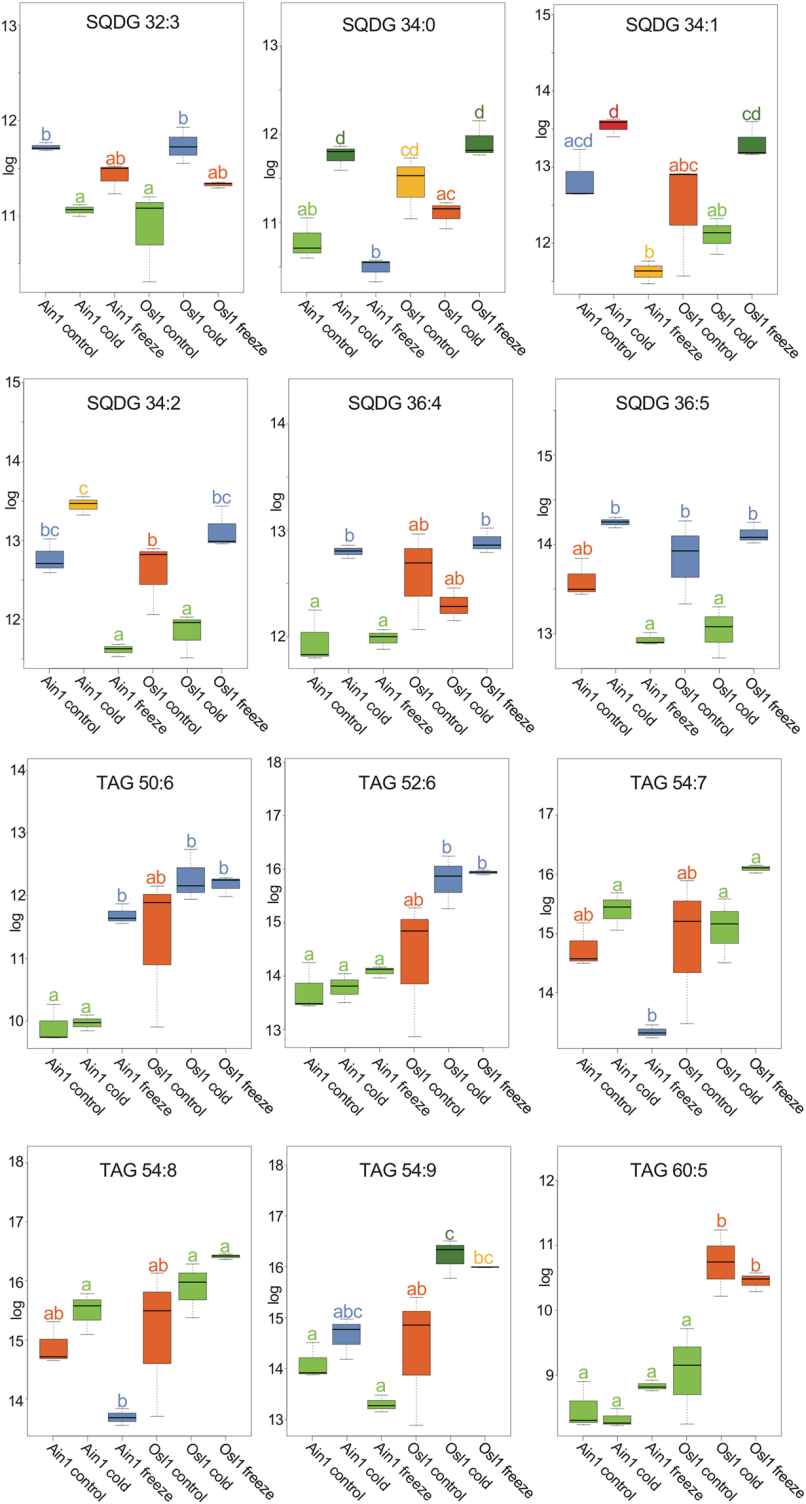
Significantly changing SQDG and TAG lipids. Illustrated are the levels of the eight significantly changing sulfoquinovosyldiacylglycerols (SQDG) and the triacylglycerides (TAG) identified via analysis of variance in two *Brachypodium sylvaticum* accessions across different temperature treatments. X-axes represent the log of relative content, y-axes represent different accessions and their respective treatments. Values are the log(mean) ± SE (n = 3). Different letters and color codes indicate significant differences (p ≤ 0.05) according to posthoc Tukey’s test.

Posthoc Tukey test showed that sample means of metabolites (Fig. 3) and lipids (Fig. 4 and Supplementary Fig. 3a–c) were accession- and treatment-specific, e.g. for erythritol two significantly different means were identified – the lower mean level incorporating Ain1 control, cold, freezing, and Osl1 cold (Fig. 3 - Tukey test group a) and the significant different higher mean containing Osl1 control and freezing samples (Fig. 3 - Tukey test group b). GABA revealed a decrease from control conditions to cold conditions in both accessions followed by a significant increase during freezing conditions. Galactinol showed higher levels in Osl1 and a general increase between control to freezing conditions in both accessions. Higher levels of erythritol and galactinol in the freezing tolerant accession supported the notion of sugar alcohols acting as agents against oxidative and osmotic stress^32,33^. Hydroxyproline and ornithine were also higher in Osl1 than in Ain1. While hydroxoproline is an essential element of the cell wall glycoprotein RSH^34^, elevated levels of ornithine have shown to alleviate the plant from abiotic stresses^35^. Glycine and tyramine, on the other hand, showed generally higher means levels for Ain1 samples and a decrease between control to freezing conditions in both accessions. The sugar maltose showed increased levels during freezing conditions in both accessions, while shikimate demonstrated a steep decrease during cold and freezing stress (in both accessions). The observation of metabolite specificity to accession and treatments supports the notion of the intertwined metabolic network, where metabolites are involved in many different metabolic pathways regulating various cellular aspects^36,37^.

For the 53 significantly changing lipids repetitive patterns were detected for the sulfoquinovosyldiacylglycerols (SQDG) and the triacylglycerides (TAG) (Fig. 4). For the SQDGs elevated levels were observed during Osl1 frozen, while for almost all frozen Ain1 a steep decrease was visible in comparison to Ain1 cold. The TAGs demonstrated an upward trend between Ain1 control versus frozen Ain1 and Osl1 control versus frozen Osl1. The finding of higher levels of SQDGs and TAGs during Osl1 freezing conditions was suggestive of their essential role for conserving membrane integrity and preventing ion leakage during cold and freezing^38^.

### PCA emphasizes differences between Osl1 and Ain1 accessions

**Figure 5:**
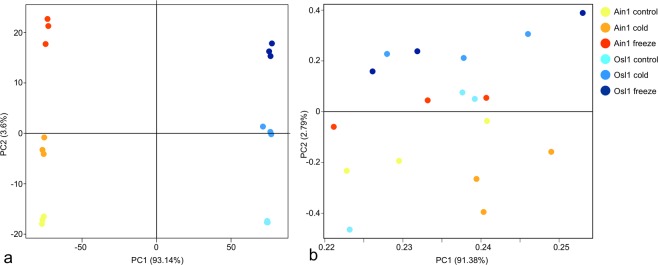
Principal component analysis (PCA) of gene expression and metabolite profiles. (**a**) PCA plot of *Brachypodium sylvaticum* accessions samples according to their normalized gene counts after variance-stabilizing transformation. The first (PC1) and second (PC2) principal components are illustrated. Samples correspond to three biological replicates from two accessions (Ain1 and Osl1) and three different temperature regimes were analyzed - represented by different color-codes; (**b**) PCA plot of metabolite profiles of corresponding samples shown in (**a**).

For the subsequent analysis, the metabolite and lipid profiles were combined into one single dataset (from here on onwards we will refer to the combined dataset as the compounds dataset). Principal component analysis (PCA) was applied to the 500 most variable genes of the normalized gene expression dataset^31^ and to the entire compounds dataset. For both PCAs, principal component (PC) 1 accounted for more than 90% of the variation observed (Fig. 5), while PCs2 accounted for 3.6% and 2.79% of the observed variation. For the PCA of the gene expression a clear separation between accessions was visible on PC1, while PC2 demonstrated a separation between treatments (Fig. 5a). The PCA based on the compound profiles revealed a gradual transition between samples on PC1 (Fig. 5b) reflecting the specificity of metabolites across treatments, as observed during the analysis for metabolic changes (see above). The loadings of PC1 revealed that while mainly metabolites could be identified on the negative end, solely lipids dominated the positive end, indicating a negative correlation between lipids and metabolites. On PC2 of the compound PCA (Fig. 5b), on the other hand, a separation between the accessions Ain1 and Osl1 was visible. Here, the positive loadings were also dominated by the lipids, but several metabolites could be detected amongst them. Particularly TAGs and hexacosanoate, ornithine, GABA, proline, hydroxyproline, erythritol, galactinol, and melibiose recorded similar loadings, indicative of a positive correlation between them.

### Correlation-based network analysis emphasizes functional group of lipids in Osl1

**Figure 6:**
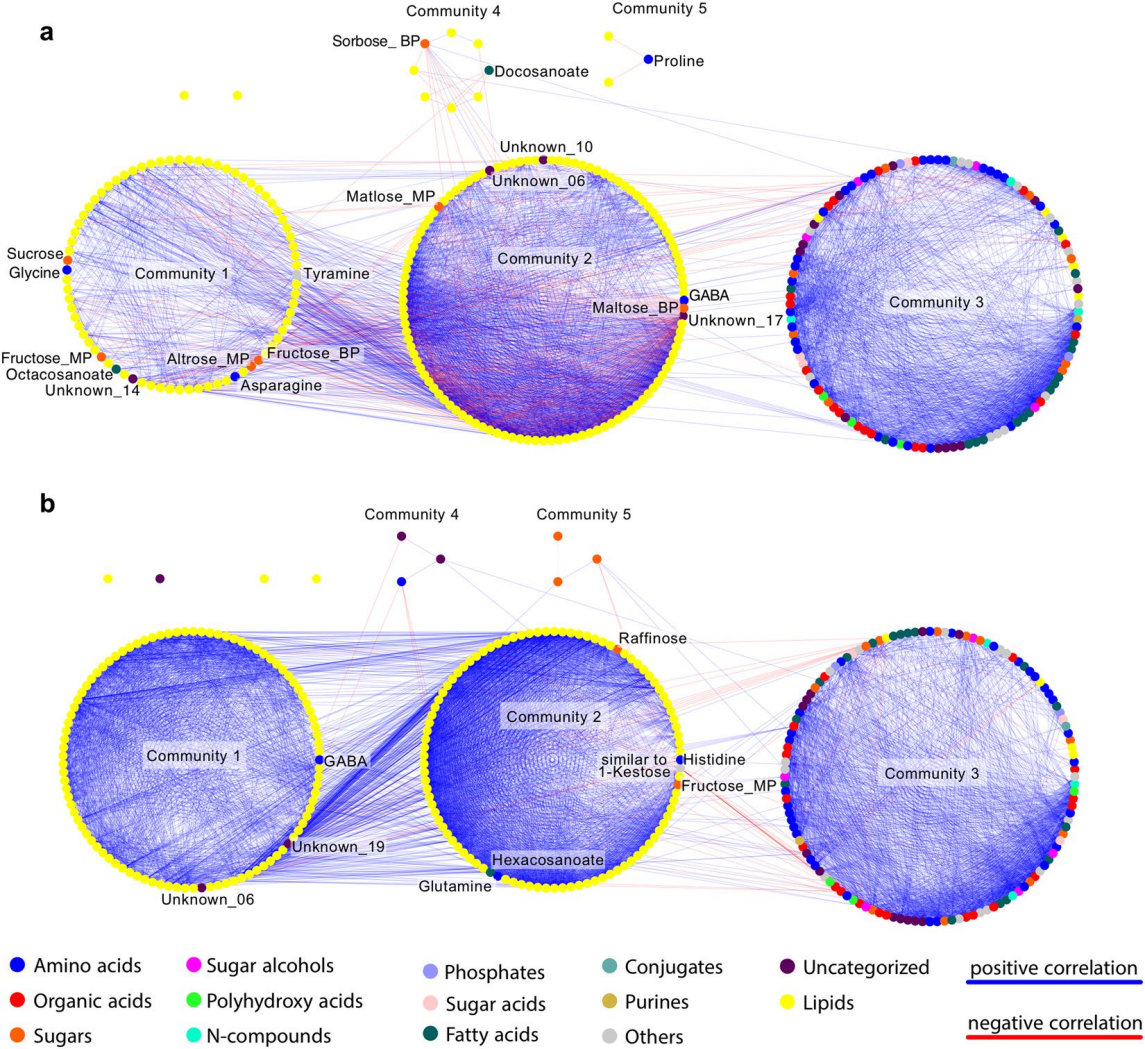
Correlation-based networks of Ain1 and Osl1. Network visualization of pairwise correlations of metabolites and lipids. The Pearson correlation was used to estimate correlation coefficients. Threshold tests for correlation coefficients (*r*) and *p*-values were applied to detect significant correlations. Thresholds were set tor *r* ≥ 0.8 and *q* of ≤0.05. Spurious correlations were removed, while significant correlations were transformed into network form. Metabolites are displayed as elliptical nodes and color-coded according to the compound classification; lipids are displayed as yellow elliptical nodes. Positive correlations are illustrated as blue edges, negative correlations as red edges. Communities in the networks were determined by the walktrap community detecting algorithm. (**a**) *B. sylvaticum* Ain1 correlation network; (**b**) *B. sylvaticum* Osl1 correlation network.

Following the separation derived from the PCA, a correlation-based network (CN) was constructed for each Ain1 and Osl1 compounds datasets^39^. In the CN, the nodes represent compounds and the edges between them the significant correlation coefficients (*r*). Thresholds for the Ain1 and Osl1 CNs were set to *r ≥ *0.8 and a q-value ≤ 0.05 to remove spurious correlations. The Ain1 CN was composed of 334 nodes, corresponding to the compounds in the dataset, and 4,538 edges, of which 3,995 corresponded to positive correlations and 543 to negative correlations, resulting in a positive edges to negative edges (pe/ne) ratio of 7.36. The Osl1 CN contained 334 nodes and 6,317 edges, of which 6,230 corresponded to positive correlations and 87 to negative correlations, resulting in a pe/ne ratio of 71.6. Due to the greater number of edges in the Osl1 network, it exerted greater values for the edge density (Ain1 = 0.08, Osl1 = 0.11) and the average weighted degree of the network (Ain1 = 24.24, Osl1 = 33.53). Clustering of metabolites in a CN into communities may identify functional groups of nodes of similar chemical properties or groups of metabolites that are tightly connected due to shared regulatory processes^26,39,40^. Nodes in the network were clustered together using the walktrap community detecting algorithm^41^, revealing four lipid communities and one community containing metabolites in the Ain1 network (Fig. 6a) and two lipid communities and three metabolite communities in the Osl1 network (Fig. 6b). Of the TAG’s identified via statistical analysis and PCA, in the Ain1 network TAG’s 50:6, 54:7, 58:8, and 54:9 clustered into community 1, while TAG’s 52:6 and 60:5 clustered into community 4. In the Osl1 network, on the other hand, all significant TAG’s clustered into a single community, namely community 1.

**Figure 7:**
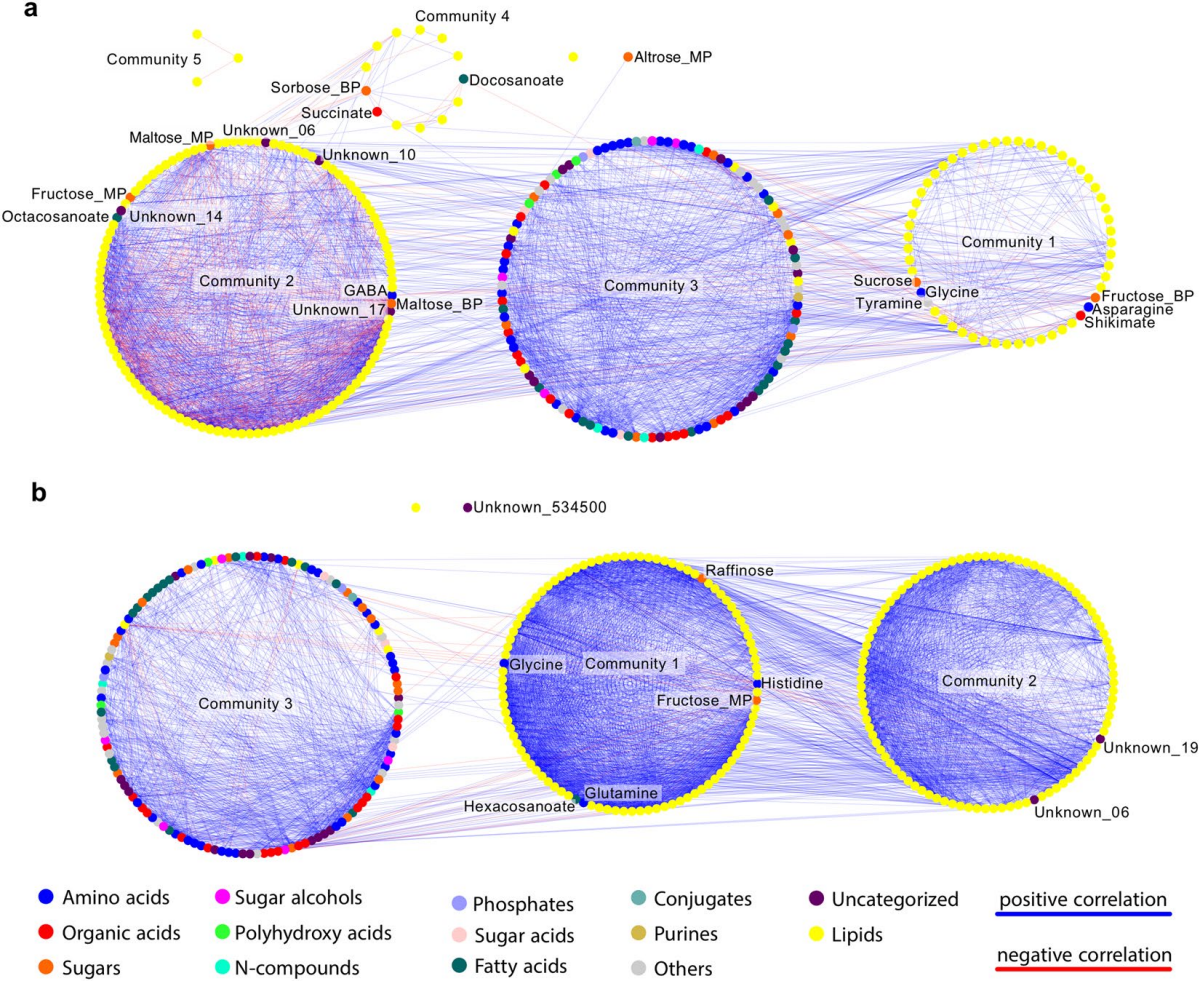
Difference set correlation-based networks of Ain1 and Osl1. Network visualization of pairwise correlations of metabolites and lipids. The Pearson correlation was used to estimate correlation coefficients. Threshold tests for correlation coefficients (*r*) and *p*-values were applied to detect significant correlations. Thresholds were set tor *r* ≥ 0.8 and *q* of ≤0.05. Spurious correlations were removed, while significant correlations were transformed into network form. Differences between networks can be determined using set theory, were the differences sets highlight nodes and edges specific to a network. Metabolites are displayed as elliptical nodes and color-coded according to the compound classification; lipids are displayed as yellow elliptical nodes. Positive correlations are illustrated as blue edges, negative correlations as red edges. Communities in the networks were determined by the walktrap community-detecting algorithm. (**a**) *B. sylvaticum* Ain1 ⊄ Osl1 difference correlation network; (**b**) *B. sylvaticum* Osl1 ⊄ Ain1 difference correlation network.

Among the lipid communities a small number of non-lipids were incorporated, mainly sugars, amino acids, fatty acids, and unknowns. To identify commonalities and differences between the networks, difference (Fig. 7a,b), intersection and symmetric difference networks (Supplementary Fig. 4a,b) were constructed. The analysis showed that 1,670 edges were common to both networks (Supplementary Fig. 4b), 2,868 out of the 4,538 edges were specific to the Ain1 network (Fig. 7a) and 4,647 out of the 6,317 edges were specific to the Osl1 network (Fig. 7b). Moreover, among all of the metabolites integrated into the lipid communities, only GABA appeared in both networks (Supplementary Fig. 4b), while altrose, maltose, sorbose, sucrose, docosanoate, octacosanoate, asparagine, and three unknowns were specific to the Ain1 network and raffinose, histidine, glutamine, hexacosanoate, and two unknowns were specific to the Osl1 network. Oligosaccharides and polysaccharides contribute to the stability of phospholipidic mono- and bilayers via their inclusion between polar headgroups^42^. However, while the sugars associated with the Ain1 network have shown to be a stabilizing agent for the plasma membrane during cold stress^43^, raffinose (Osl1 network) appeared to be restrained to the chloroplast inner membrane^44^. Nevertheless, raffinose has been demonstrated to act as a cryoprotectant in cold acclimated *Arabidopsis* ecotypes^45^. In the same study, a positive correlation between raffinose and glutamine was demonstrated. Asparagine, on the other hand, decreased during cold adaptation in the perennial ryegrass^46^. The occurrence of different long chain fatty acids in the Ain1 and Osl1 difference network may hint at the discerned regulation of lipid biosynthesis^47^.

Among the putative functional group of lipids in the Ain1 network, one positive significant connection between GABA and TAG 50:6 (correlation coefficient 0.92) and three negative significant correlations to TAG’s 54:7, 54:8, 54:9 (correlation coefficient < −0.8) were present. Furthermore, maltose maintained two significant negative connections to TAGs 54:7 and 54:8 (correlation coefficient < −0.8). In the Osl1 network, the GABA and maltose significant correlations did not appear. However, galactinol displayed a significant positive correlation to TAG 60:5, while hexacosanoate displayed significant positive correlations to all TAG’s (correlation coefficient > 0.85).

### CNA combined with machine learning predicts GABA shunt activities for Ain1

To put the metabolic and expression data into a biological context, we applied novel algorithms for the analysis of big biological data. First, we applied CNA combined with ML techniques^29^. This approach explores the fact that metabolic pathways shape the topology of a CN. By mapping known metabolic pathways into the CN and learning their topological conformation, a ML model is generated capable of predicting the presence of unknown pathways. To do so, for each CN one ML model was generated (see above and Materials and Methods for details) employing the extreme gradient boost algorithm^48^. Performance evaluation of the ML models showed an AUC of the receiving operating characteristic curve of 0.986 for the Ain1 CN model and 0.992 for the Osl1 CN model (Supplementary Fig. 5). The confusion matrix of the models demonstrated an accuracy of 0.934 for the Ain1 model and 0.921 for the Osl1 model (Table 1). The cross-validated models where then used to predict the presence/activity of 278 different metabolic pathways gathered from the PlantCyc repository^36^ at a prediction threshold value of ≥0.5. Sensitivity analysis was applied to validate the prediction values of the metabolic pathways. Only pathways that tested positive during prediction and sensitivity analysis were considered positively predicted. The analysis suggested the presence of five pathways that occurred in Ain1 and Osl1, 15 pathways specific to Ain1, and 19 pathways specific to Osl1 (Table 2). Pathways specific to Ain1 centered mainly on the amino acids GABA, glutamate and the organic acids fumarate, succinate, 4-OH-benzoate, and the N-compound putrescine, i.e. 4-aminobutanoate degradation I and IV, L-glutamate degradation IV, putrescine degradation IV, and the reductive TCA cycle I. TCA cycle intermediates, putrescine, glutamate, and GABA are inherently related, such that 2-oxoglutarate is converted via glutamate dehydrogenase (E.C. 1.4.1.3) to glutamate which in turn is decarboxylated via glutamate decarboxylase (E.C. 4.1.1.15) to GABA. Via the conversion of GABA to succinate semialdehyde mediated by 4-aminobutyrate-aminotransferase (E.C. 2.6.19) and subsequently to succinate via succinate semialdehyde dehydrogenase (E.C. 1.2.1.24), the GABA shunt is completed. Moreover, GABA can be produced using putrescine, which is oxidized by diamine oxidase (E.C. 1.4.3.22) to 4-amino-butanal and then catalyzed to GABA via aminobutyraldehyde dehydrogenase (E.C. 1.2.1.19). The pronounced occurrence of glutamate, GABA, putrescine and TCA cycle intermediates was indicative for the upregulation of the GABA shunt, which is known for sustaining the TCA cycle under abiotic and biotic stress conditions^49–51^. The relation between the GABA shunt and the TCA cycle was supported by identification of the reductive TCA cycle I pathway specific to Ain1 (Table 2). Other noteworthy metabolic pathways detected for Ain1 were free phenylpropanoid acid biosynthesis based on cis- and trans-sinnnapate and 4-OH-cinnamate, fatty acids biosynthesis based on malonate and adenosine-5-monophosphate, and photorespiration based on glycerate, glycerate-3-phosphate, glutamate, serine, glycine, and 2-oxoglutrate.

**Table 1:** Confusion Matrices of Ain1 and Osl1 ML models.

		Ain1 ML model		Osl1 ML model	
		Actual class		Actual class	
Predicted class		1	2	1	2
	1	38	5	38	6
	2	0	33	0	32

1 = positive instances, 2 = negative instances.

**Table 2:** Prediction table.

	Metabolic pathways	Predictionvalue	Sensitivity analysis
Ain1 + Osl1	gamma-glutamyl cycle	0.74, 0.96	0.71, 0.92
	glutathione degradation	0.74, 0.96	0.71, 0.92
	glycine biosynthesis	0.74, 0.74	0.78, 0.81
	L-asparagine biosynthesis II	0.74, 0.85	0.82, 0.87
	suberin monomers biosynthesis	0.74, 0.85	0.78, 0.85
Ain1	4-aminobutanoate degradation I	0.96	0.94
	4-aminobutanoate degradation IV	0.96	0.94
	4-hydroxybenzoate biosynthesis I (eukaryotes)	0.74	0.85
	4-hydroxybenzoate biosynthesis IV	0.59	0.53
	4-hydroxybenzoate biosynthesis V	0.59	0.53
	fatty acid biosynthesis (plant mitochondria)	0.59	0.56
	free phenylpropanoid acid biosynthesis	0.59	0.50
	homocarnosine biosynthesis	0.96	0.94
	L-glutamate degradation IV	0.96	0.93
	nitric oxide biosynthesis II (mammals)	0.93	0.92
	photorespiration	0.74	0.78
	putrescine degradation IV	0.74	0.78
	reductive TCA cycle I	0.74	0.81
	trehalose degradation II (trehalase)	0.93	0.90
	UMP biosynthesis II	0.74	0.82
Osl1	(S)-reticuline biosynthesis I	0.96	0.90
	2′-deoxymugineic acid phytosiderophore biosynthesis	0.73	0.74
	3-cyano-L-alanine + H + + 2 H2O - > ammonium + L-aspartate	0.73	0.76
	4-hydroxyphenylpyruvate biosynthesis	0.96	0.90
	ajugose biosynthesis II (galactinol-independent)	0.88	0.80
	cyanide detoxification I	0.96	0.91
	gamma-glutamyl cycle (plant pathway)	0.73	0.70
	hypoglycin biosynthesis	0.73	0.82
	L-asparagine biosynthesis I	0.73	0.80
	L-asparagine degradation I	0.73	0.76
	L-glutamine + L-aspartate + ATP + H2O - > L-glutamate + L-asparagine + AMP + diphosphate + H+	0.73	0.80
	L-phenylalanine biosynthesis III (cytosolic, plants)	0.85	0.84
	L-tyrosine biosynthesis II	0.96	0.90
	L-tyrosine biosynthesis III	0.96	0.90
	rosmarinic acid biosynthesis I	0.96	0.90
	rosmarinic acid biosynthesis II	0.96	0.90
	S-methyl-5-thio-alpha-D-ribose 1-phosphate degradation	0.73	0.79
	stachyose biosynthesis	0.73	0.71
	superpathway of phospholipid biosynthesis II (plants)	0.96	0.87

Pathways that were specific to Osl1, centered mainly on the amino acids asparagine, aspartate, cysteine, and tyrosine and the organic acid 2-oxo-glutarate, suggestive for ammonium and cyanide metabolism. Cyanogenic plants liberate cyanide from cyanogenic glucosides and lipids when they are in contact with predatory herbivores^52^. Also, accumulation of hydrogen cyanide, as a co-product of ethylene biosynthesis, has been reported in sorghum and barley during abiotic stresses, such as water-deficit^53^ and salinity^54^.The accumulation of hydrogen cyanide in plants has been associated with cell signaling^55^ and nitrogen-recycling processes^54^. However, too high levels of hydrogen cyanide may result in plant death as for instance caused by bacteria^56^. In this regard, the occurrence of the cyanide detoxification I pathway in Osl1 may render beneficial to counteract cyanide contamination under freezing stress. Also, the superpathway of phospholipid biosynthesis based on serine and myo-inositol was predicted for Osl1. This pathway is one of the main producers of phospholipids for membranes in plants^57^ and may provide the means to sustain membrane integrity in Osl1. Other noteworthy pathways specific to Osl1 were stachyose biosynthesis based on galactinol, sucrose, raffinose and myo-inositol and ajugose biosynthesis based on raffinose and sucrose.

### Genetic algorithm (GA) identifies accession-specific transcription factors and acetyltransferases

**Figure 8:**
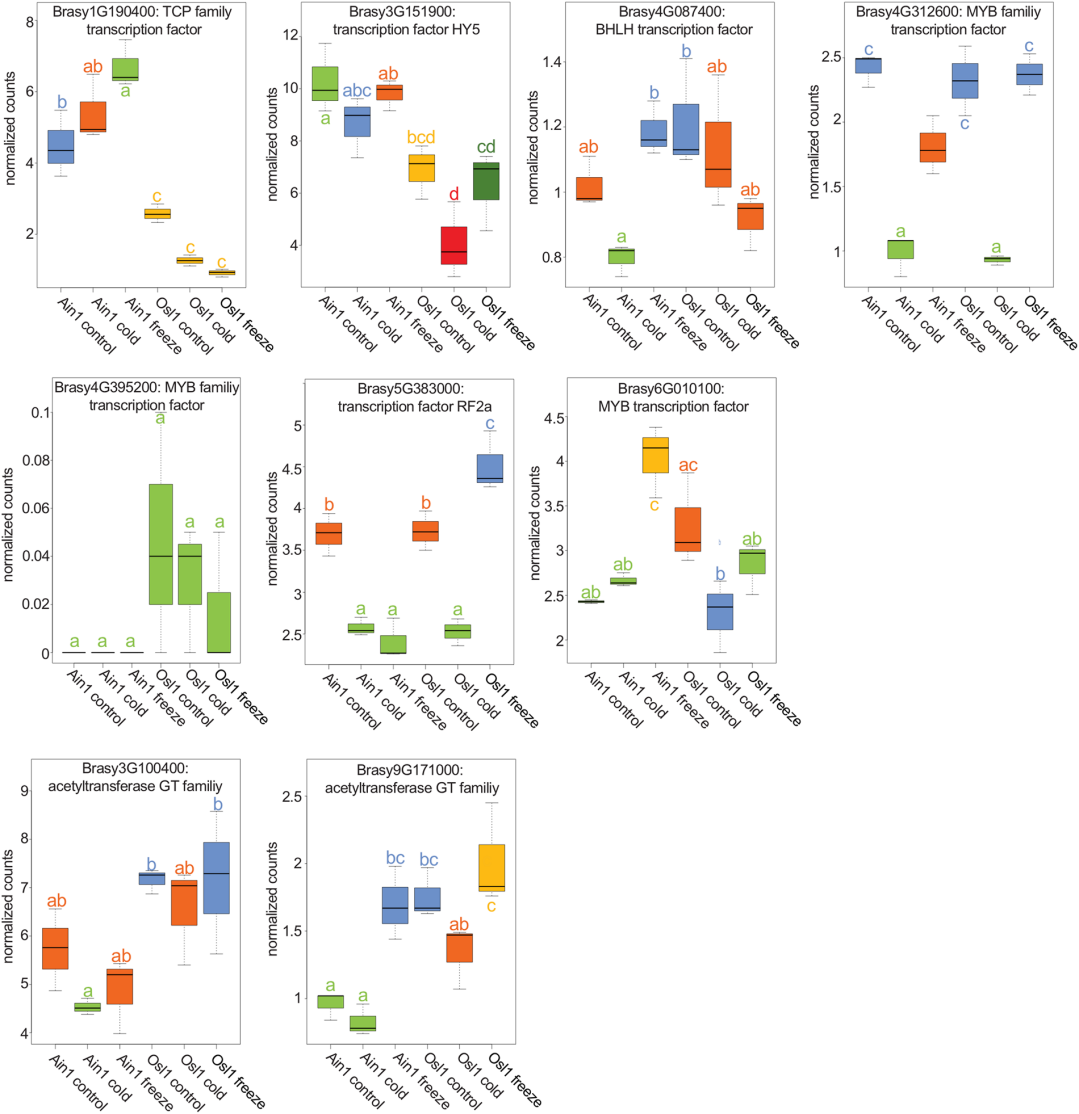
Transcription factors and acetyltransferases. Illustrated are the levels of the seven transcription factors activity associated genes (rows 1 and 2) and two acetyltransferases (row 3) identified via a genetic algorithm in two *Brachypodium sylvaticum* accessions across different temperature treatments. X-axes represent the normalized gene counts, y-axes represent different accessions and their respective treatments. Values are the mean ± SE (n = 3). Different letters and color codes indicate significant differences (p ≤ 0.05) according to posthoc Tukey’s test.

To integrate the gene expression data to the metabolic pathway analysis, we employed a GA that optimizes the correlation between a trait of interest and a subset of genes within a gene co-expression network, resulting in a number of genes that is sufficiently low to facilitate their analysis^30^. We made adequate modifications to the GA in order to correlate groups of metabolites representing the metabolic pathways identified in the previous analysis rather than single traits (see Materials and Methods). Correlations were estimated at absolute values so that negative correlating genes were included in the resulting set of genes. As a result of the previous analyses pointing to GABA and organic acids in relation to lipid regulation in Ain1, we investigated pathways associated with GABA metabolism (4-aminobutanoate degradation I and IV) and the TCA-cycle. For the metabolites associated with the 4-aminobutanoate degradation I pathway, 74 genes were identified (Supplementary Data S2). The correlation coefficient between the first principal components of the metabolites and genes was computed with 0.698. The average expression of the 74 genes showed similar patterns in Ain1 and Osl1 (Supplementary Fig. 6), revealing decreasing values from control to cold conditions followed by a significant increase under freezing. However, the greatest average values were achieved during Ain1 freezing, indicating increased transcription of genes associated with GABA, alanine, succinate, metabolism. A similar picture arose for 4-aminobutanoate degradation VI (GABA, alanine, succinate, pyruvate, 2-oxoglurate, and glutamate), where 108 genes were identified with a correlation coefficient of 0.796. For the L-glutamate degradation IV pathway (2-oxo-glutrate, alanine, glutamate, GABA, pyruvate, and succinate), 104 genes were detected with a correlation coefficient of 0.619. For the putrescine degradation IV pathway (putrescine and GABA), 112 genes were found with a correlation coefficient of 0.979. Metabolites associated with the reductive TCA cycle 1 (citrate, fumarate, succinate, pyruvate, adenosine-5-monophosphate, 2-oxoglutrate) were also analyzed. Here, 461 genes were detected and the correlation coefficient was estimated at 0.859. The average expression patterns demonstrated a similar pattern as before (Supplementary Fig. 6). Examination of the identified genes, highlighted two acetyltransferases of the Gcn5-related N-acetyltransferases family (GNAT)^58^, 10 transcription factor-associated genes, and 14 genes encoding membrane proteins. GNAT enzymes catalyze the transfer of an acyl moiety from acyl coenzyme A (acyl-CoA) to a large number of different substrates and are essential for lipid biosynthesis^59^. Here, the two acetyltransferases (*Brasy3G100400* and *Brasy9G171000* – Fig. 8) showed increased transcription in Osl1 and particularly in Osl1 under freezing. Out of the 10 transcription factor-associated genes, seven genes displayed Ain1- or Osl1-specific expression patterns. *Brasy1G190400* (TCP family transcription factor), *Brasy3G151900* (transcription factor HY5), *Brasy4G087400* (BHLH transcription factor), *Brasy6G0*1*0100* (MYB transcription factor) were, in general, more abundant in Ain1 and increased during freezing conditions, while *Brasy4G31*2*600*, *Brasy4G395200* (MYB family transcription factors) and *Brasy5G38300* (RF2a transcription factor) were more abundant in Osl1 (Fig. 8). The distinct patterns of transcription associated with GABA and TCA-cycle intermediate metabolism and with transcription factors and acetyltransferases hinted at accession-specific regulation of lipid biosynthesis.

## Discussion

We characterized the responses of two *B. sylvaticum* accessions, isolated from two geographical areas with contrasting climates (i.e., Osl1 from North Europe and Ain1 from North Africa – Fig. 1). Our aim was to characterize freezing tolerance mechanism(s) in this model perennial grass that may contribute into unraveling similar mechanisms in biomass crops such as switchgrass and *Miscanthus*^2,60^. Osl1 plants displayed higher freezing tolerance than Ain1, as indicated by increased vigor, higher chlorophyll contents, lower MDA, lower ion leakage and higher PSII protein accumulation (Fig. 1).

Next, we profiled all samples for gene expression and metabolites and lipids (compounds dataset). PC1 of the gene expression PCA showed a clear separation between accessions and separation of treatments on PC2 (Fig. 5a). The PCA of the compounds dataset showed a transitional change between treatments on PC1 and a separation between accessions on PC2 (Fig. 5b). The conclusions of the PCA were twofold: i) the differences observed on a transcriptional and metabolic level were mainly due to genotypic differences ii) posttranscriptional and posttranslational events were highly regulated, resulting in transitional rather than separational metabolic phenotypes between Ain1 and Osl1. Differential gene expression analysis revealed increased changes of gene expression in Ain1 in comparison to the freezing adapted Osl1. A lower degree of adjustment of transcription supported the notion of Osl1 adaptability to freezing stress by showing lower levels of response^61,62^. We constructed correlation-based networks for Ain1 and Osl1^39^. Intersection, differences, and symmetric differences highlighted the topological differences between the networks (Figs. 6 and 7 and Supplementary Fig. 4). The higher network connectivity of the cold-adapted accession Osl1, together with a higher ratio (~10 fold) of positive to negative correlations, supported the ability of Osl1 to adapt its metabolism at the initiation of the abiotic stress, similar to that observed in grapevine leaves^63^ or tomato seeds^64^.

Overwintering plants have evolved various strategies to minimize freezing injuries. These include the modification of membrane fatty acid composition, accumulation of carbohydrates and other compatible solutes and the production of antioxidants and antifreeze proteins^65^. Our statistical, multivariate, and correlation-based network analyses emphasized the role of GABA, glutamate, putrescine, TCA cycle intermediates, and SQDGs and TAGs lipids. While the link between GABA and the TCA cycle, and its involvement in abiotic stress, is well known^66,67^, we were particularly interested in their roles with respect to lipid biosynthesis and their potential impact on the membrane integrity of the two *B. sylvaticum* accessions. We employed state-of-the-art algorithms that place big biological data into biological context by combining CNA with machine-learning techniques. The idea behind this approach is that the computer learns from existing data. Based on recurring patterns that known metabolic pathways imprint onto correlation networks, the computer is able to predict the existence or ‘activity’ of unknown metabolic pathways. The approach has proven so powerful that it was able to predict the melibiose degradation pathway in tomato, although melibiose was not part of the original dataset^29^. Rooted on the two correlation-based networks, we generated two ML models (ML-Ain1 and ML-Osl1 – Supplementary Fig. 5) and then predicted the ‘activity’ of metabolic pathways as annotated in the PlantCyc collection^36^. While the ML-Ain1 model was primarily associated with GABA metabolism and the reductive TCA cycle, these pathways were not predicted by the ML-Osl1 model (Table 2), indicative of their downregulation. We also employed a genetic algorithm that maximized the correlation between a subset of genes in a gene expression network and a trait of interest^30^ – in this case, sets of metabolites corresponding to metabolic pathways. The analysis highlighted a TCP family and the HY5-transcription factors showing elevated levels for Ain1 in comparison to Osl. Also two acetyltransferases were identified with higher expression levels for Osl1 (Fig. 8).

The association between GABA and the TCA cycle intermediates is to replenish the TCA cycle via the GABA shunt and to maintain amino acid biosynthesis under low levels of energy^49,50,67^. GABA accumulation has been associated with abiotic stresses^50,68^. Increased GABA levels were shown in wheat and barley during freezing^69^. In our study, significant GABA increases were seen in both Ain 1 and Osl1 under freezing (Fig. 3). Nevertheless, we could predict GABA-associated pathways only in Ain1, indicating that under freezing Ain1 activated the GABA shunt, but not Osl1. Given the sensitivity of Ain1 to freezing, the activation of the GABA shunt may have had detrimental effects in Ain1. In transgenic *Arabidopsis* seeds the accumulation of high levels of GABA impaired fatty acid biosynthesis^70^. Bypassing the TCA cycle via the GABA shunt results in decreased levels of CoA moieties production, essential for fatty acid biosynthesis. Moreover, high levels of GABA have been associated with low levels of malate, which has been reported to be the preferred fatty acid elongation substrate^71,72^. In yellow lupine seedlings, C atoms from acetate may be preferably incorporated into amino acid synthesis rather than fatty acid synthesis^73^ and similar mechanisms may play a role in reducing fatty acid biosynthesis leading to membrane leakage under freezing conditions in Ain1. In Osl1, on the other hand, the GABA shunt activity was downregulated as a result of normal TCA cycle activities and the maintenance of fatty acid biosynthesis, which are regulated by acetyltransferases, leading to the formation of functional, non-leaky membranes.

Taken together, our top-down-based approach, employing state-of-the-art algorithms for big data, was able to filter through physiological, transcription, metabolite and lipid data to provide evidence highlighting different regulatory mechanisms taken place in Ain1 and Osl1 during freezing. While the upregulation of the GABA shunt activity showed detrimental effects on fatty acid biosynthesis, Osl1 downregulated the GABA shunt activity to counteract the inhibition of fatty acid biosynthesis. Consequently, membrane leakage was reduced contributing to the survival of Osl1 plants under freezing conditions.

## Methods

### Plant material, growth conditions, and experimental design

Seeds from *B. sylvaticum* accessions Ain1 and Osl1 were obtained from the USDA National Plant Germplasm System (https://www.ars-grin.gov/npgs/). For the cold experiments, the seeds were stratified in a cold room at 10 h/1 h light/dark cycles at 4 °C for 2 weeks before sown into soil. Germination and growth were achieved in a chamber room 16 h/8 h light-dark cycles at 26 °C/18 °C, respectively, for 3 weeks. For cold acclimation, the plants were moved into a cold room at 10 h/14 h light/dark cycles at 4 °C, for 3 weeks.

The freezing experiments were conducted in a temperature and light controlled growth chamber. The initial 24 h were composed of an 8 h light period at 4 °C, followed by a dark period of 2 h at 4 °C, 30 min at 2 °C, 30 min at 0 °C, 4 h at −2C, 8 h at −5.5 °C, 30 min at −2 °C, and 30 min at 1 °C. For the subsequent 24 h, plants were kept at dark at 4 °C. After the freezing treatment, all plants were moved to growth chambers where they were kept at 16 h/8 h light-dark cycles at 26 °C/18 °C, respectively.

Leaf samples of seedlings were collected i) before the cold acclimation (CK) treatment was initiated, ii) before the freezing challenge was initiated, and iii) after 8 h of freezing at −5.5 °C. All samples were subjected to RNA and metabolite extraction for subsequent gene expression and metabolomics analysis, respectively (see below). Ion leakage measurements and immunoblot analysis were performed immediately after freezing challenge using control plants as a reference control. The plant damage phenotyping and sampling for chlorophyll and MDA contents were performed after 2 weeks of recovery in growth chamber rooms using control plants as a reference control.

### Electrolyte leakage

Shoot samples for electrolyte leakage were harvested immediately after freezing challenge. Electrolyte leakage was measured as described previously^74^.

### Inmunoblot analysis

Plant leaf tissues were weighed, frozen in liquid N_2_, and ground in three volumes of 2 × Laemmli sample buffer. Total proteins were separated by SDS-PAGE, transferred to a polyvinylidene difluoride membrane (Bio-Rad), and probed as described previously^75^. Antibodies raised against PsbA/D1 (AS01016), and Lhcb2 (AS01003) and PsbO 1 (AS05092), were obtained from Agrisera (Vannas, Sweden). Horseradish peroxidase-conjugated secondary antibodies were purchased from Santa Cruz Biotechnology.

### Chlorophyll measurements

Shoots were grinded in liquid N_2_ and weighed. Chlorophyll was extracted in 80% acetone, and the absorbance was measured at 663 nm and 645 nm (Synergy™ Mx Microplate Reader; BioTek, USA). Total chlorophyll content was calculated as described elsewhere^76^.

### Malondialdehyde measurements (MDA)

Shoots were grinded in liquid N_2_ and weighed. Tissue was homogenized with 5 ml of 50 mM NaH_2_PO_4_-Na_2_HPO_4_ buffer pH 7.5 and centrifuged at 20,000 g for 25 min. For MDA measurements, 4 ml of 20% trichloroacetic acid containing 0.5% thiobarbituric acid were added to a 1 ml aliquot of the supernatant. The mixture was heated at 95 °C for 30 min, quickly cooled in ice, and then centrifuged at 10,000 g for 10 min. The absorbance of the supernatant was measured at 532 nm and 600 nm^77^.

### Metabolite profiling by gas chromatography‒mass spectrometry

Metabolites were extracted using the MTBE method as described elsewhere^78^. One hundred-fifty μl vacuum-dried polar phases samples were derivatized and subjected to GC‒MS analysis as described previously^79^. The GC‒MS data were obtained using an Agilent 7683 series auto-sample (Agilent Technologies, http://www.home.agilent.com), coupled to an Agilent 6890 gas-chromatograph‒Leco Pegasus two time-of-flight mass spectrometer (Leco: http://www.leco.com/). Identical chromatogram acquisition parameters were applied to those previously used^80^. Chromatograms were exported from the LECO CHROMATOF software (version 3.34) to the R software. Ion extraction, peak detection, retention time alignment and library searching were obtained using the TargetSearch package from Bioconductor^81^. The resulting data matrix was used for further analysis.

### Lipid profiling

Lipids were extracted using the MTBE method as described previously^77^. Five hundred μl vacuum-dried organic phase samples were processed using ultra-performance liquid chromatography, on a C8 reverse phase column coupled with Fourier transform mass spectrometry (UPLC-FT-MS) (Thermo-Fisher, http://www.thermofisher.com) in positive and negative ionization modes. Processing of chromatograms, peak detection and integration were performed using REFINER MSH 10 (GeneData, http://www.genedata.com). Processing of mass spectrometry data included the removal of the fragmentation information, isotopic peaks and chemical noise. Selected features were annotated using an in-house lipid database^80^.

### Statistical analysis of metabolite and lipid dataset

All metabolite and lipid data was natural-log transformed. Missing values (4.4%) were imputed using data imputation provided by Bioconductor (http://www.bioconductor.org/) package pcaMethods^82^. Analysis of variance (ANOVA) was run for each metabolite and lipid. To detect significant differences, p-values (threshold ≤ 0.05) were *Bonferroni* corrected to account for multiple hypothesis testing. Subsequently, a posthoc Tukey test was performed.

### Library preparation and sequencing

RNA was extracted with Trizol (Invitrogen) and PureLink RNA Mini Kit (Ambion) and then treated with TURBO DNase (Ambion). The RNA quality control was accessed using 2100 Bioanalyzer and RNA 6000 Nano Kit (Agilent), Qubit RNA BR Assay Kit (Thermol Fisher Scientific) and Nanodrop (Thermol Fisher Scientific). RNA-sequencing libraries were constructed using TruSeq Stranded mRNA Library Preparation Kit (Illumina). PolyA + RNA was isolated from 1 μg total RNA and then fragmented. The cDNA was synthesized, adenylated at 3′ ends and ligated with adaptors. The libraries were amplified with 8 cycles of polymerase chain reaction (PCR). Sequencing was done on a Illumina Hiseq platform of 2 × 150 bp. Filtered reads from each library were aligned to the reference genome using HISAT version 0.1.4-beta^83^, featureCounts was used to generate the raw gene counts. Gene counts were normalized using DESeq. 2 (version 1.8.1)^31^ DESeq. 2 was also used to determine differentially expressed genes between pairs at a *q*-value of ≤0.05.

### PCA and correlation-based network analysis

Principal component analyses were performed on the metabolite combined with the lipid dataset as well as on the gene expression datasets to determine clusters of associated samples. For the identified clusters, metabolite correlation networks (CN) were constructed as described before^39^. In brief, all metabolites were pairwise-correlated using Pearson correlation. Threshold tests for correlation coefficients (*r*) and *p*-values were applied to detect significant correlations. The threshold for *r*-values was set to ≥0.8 for the Ain1 and Osl1 network, respectively. A false discovery rate^84^ was applied to adjust for multiple hypothesis testing at a *q*-value of ≤0.05. Spurious correlations were removed, while significant correlations were transformed into network form.

### Identification of metabolic pathways within CN’s

Identification of metabolic pathways within CN’s was performed essentially as described in^29^. The approach employs machine-learning techniques to train a model based on known metabolic pathways and then predicts the presence of pathways. The applied method uses pathways based on the PlantCyc repository (https://plantcyc.org/)^36^ However, a collection of metabolic pathways associated with *B. sylvaticum* has yet to be established. Therefore, for the current study, a consensus set of pathways found in all 101 plant species listed in PlantCyc version 13 was generated, detecting 38 consensus pathways (positive instances). The consensus pathways were mapped to the CN’s upon which for each pathway a set of network features was computed = positive training set^29^. As a negative training set 19 random pathways (negative instances) from MetaCyc collection (https://metacyc.org/)^85^ that could not be found within the PlantCyc collection but could be mapped to the CN’s were selected. In addition, 19 random subsets of metabolites were generated accounting for the other half of the negative training set. Also here, for each instance of the negative training set the aforementioned network features were computed. To avoid over-fitting, the most important features were selected for each training-set. Next, the gradient boosting (xgboost) algorithm^48^ was employed with a 10-fold cross validation to generate ML models for the Ain1 and Osl1 networks. Predictions for all test-set metabolic pathways were generated for the final models. The quality of ML models were assessed using performance measures, i.e. the area under the curve (AUC) of the receiving operating characteristic curve, which was generated by plotting the true positive rate against the false positive rate at different prediction thresholds. An AUC close to 1 indicated a perfect classifier, while an AUC of 0.5 indicated a poor classifier that could also be achieved by chance. Another mean to demonstrate ML performances is the usage of confusion matrices, where the predicted vs. the actual classification of positive and negative instances is captured in table form. The classifications of the predicted instances correspond to the rows, while the actual classifications correspond to the columns. Positive instances are denoted as ‘1’ and negative instances are denoted as ‘2’. Derived from the confusion matrix is the accuracy measure, which is the proportion of correctly classified positive and negative instances. Finally, sensitivity analyses were performed based on the final models, where subsets (80%) of the training-instances were randomly chosen to recreate models with identical settings. Sensitivity analyses were performed to test for the robustness of the ML model. After each model generation, the test-set instances were subjected to prediction. This analysis was performed with 100 repetitions, after which the average prediction values and their corresponding variance values were computed. Test-set pathways that demonstrated prediction values ≥ 0.5 in the original model as well as in the average values of the sensitivity analysis were considered validly predicted.

### Genetic algorithm

To identify genes associated with metabolic pathways, a genetic algorithm to optimize the correlation between a trait of interest and a subset of genes within a gene co-expression network trait to gene was used as described previously^30^. The genetic algorithm was adapted so that essentially:

$$ARGMA{X}_{C}\{|cor(prcomp(Expr[\,\cdot ,C]),prcomp(Pr[\cdot ,M]))|\}$$

where *C* is a subset of genes in the module, *Expr*$[\cdot ,C]$ is the expression matrix narrowed down to *C*, *M* is a subset of metabolites corresponding to the metabolic pathway, *Pr*$[\,\cdot ,M]$ is the metabolite profile matrix narrowed down to *M*, *prcomp* is the 1^st^ principal component of a matrix, and *cor* is the Pearson correlation.

## Supplementary information

Supplementary figure 1.

Supplementary figure 2.

Supplementary figure 3a.

Supplementary figure 3b.

Supplementary figure 3c.

Supplementary figure 4.

Supplementary figure 5.

Supplementary figure 6.

Supplementary figure 7.

Supplementary data S1.

Supplementary data S2.

## Data Availability

Data associated with the current study can be found under: https://figshare.com/account/home#/projects/68999.
