# Supplementary figures and images for: Correlation-based network analysis combined with machine learning techniques highlight the role of the GABA shunt in Brachypodium sylvaticum freezing tolerance
Source: Sci Rep. 2020 Mar 11;10:4489. doi: 10.1038/s41598-020-61081-4 (PMC7066199; doi:10.1038/s41598-020-61081-4)

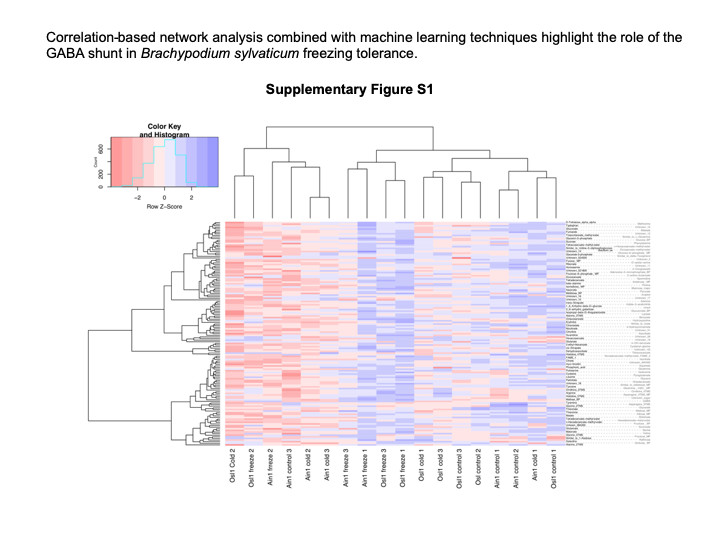

Supplement: Supplementary file 1 — Supplementary figure 1. [file 41598_2020_61081_MOESM1_ESM.tiff]

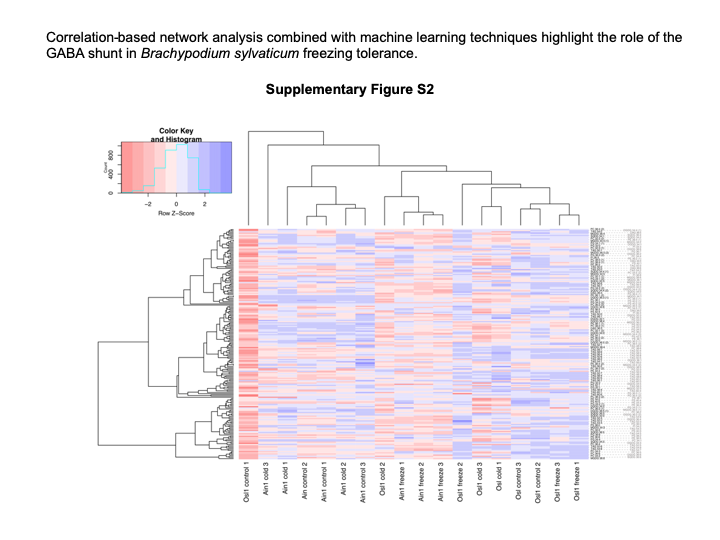

Supplement: Supplementary file 2 — Supplementary figure 2. [file 41598_2020_61081_MOESM2_ESM.tiff]

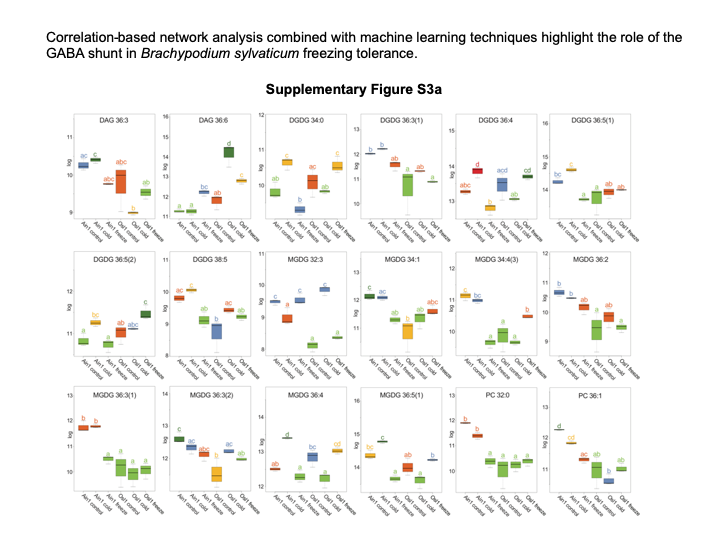

Supplement: Supplementary file 3 — Supplementary figure 3a. [file 41598_2020_61081_MOESM3_ESM.tiff]

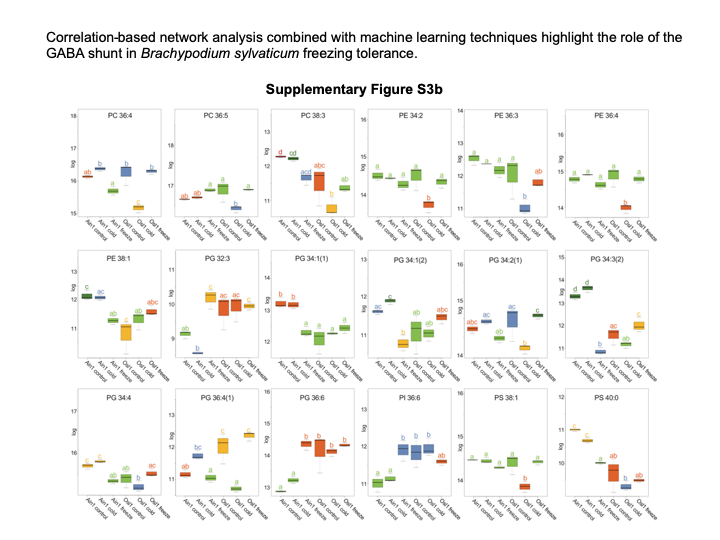

Supplement: Supplementary file 4 — Supplementary figure 3b. [file 41598_2020_61081_MOESM4_ESM.tiff]

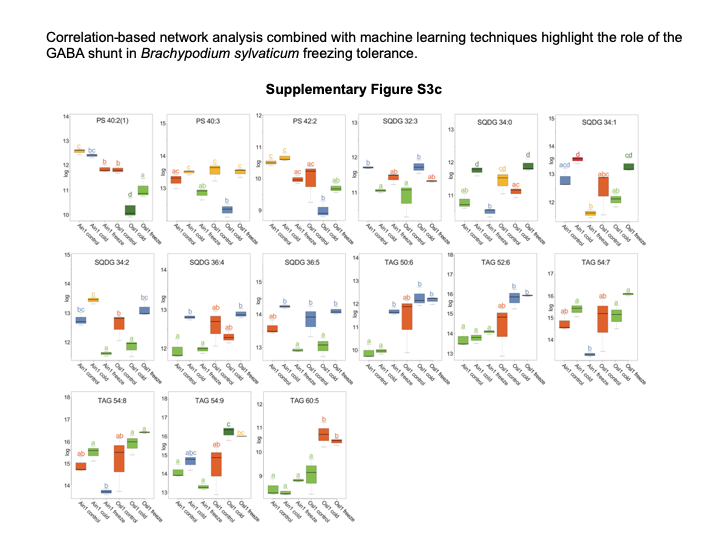

Supplement: Supplementary file 5 — Supplementary figure 3c. [file 41598_2020_61081_MOESM5_ESM.tiff]

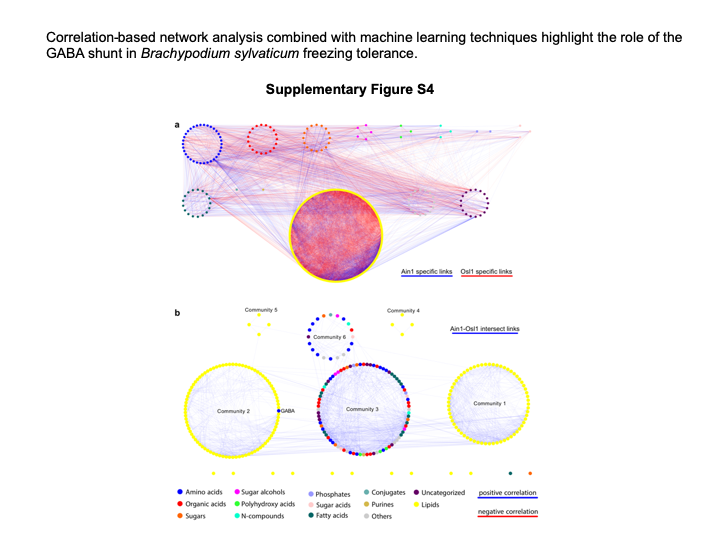

Supplement: Supplementary file 6 — Supplementary figure 4. [file 41598_2020_61081_MOESM6_ESM.tiff]

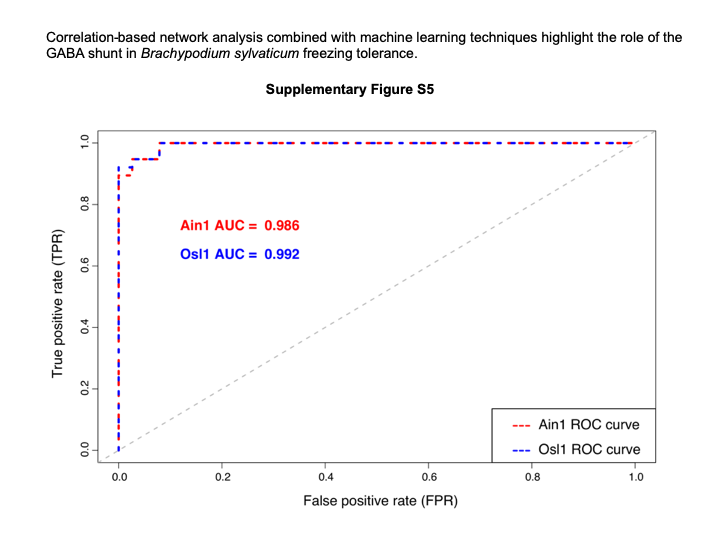

Supplement: Supplementary file 7 — Supplementary figure 5. [file 41598_2020_61081_MOESM7_ESM.tiff]

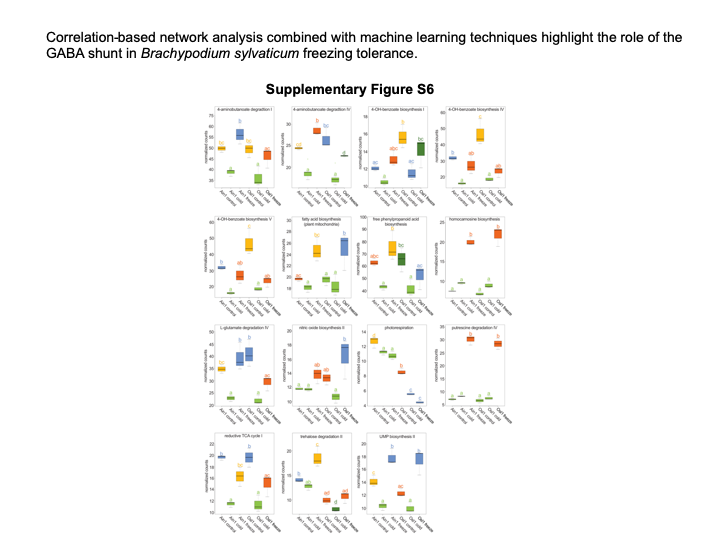

Supplement: Supplementary file 8 — Supplementary figure 6. [file 41598_2020_61081_MOESM8_ESM.tiff]

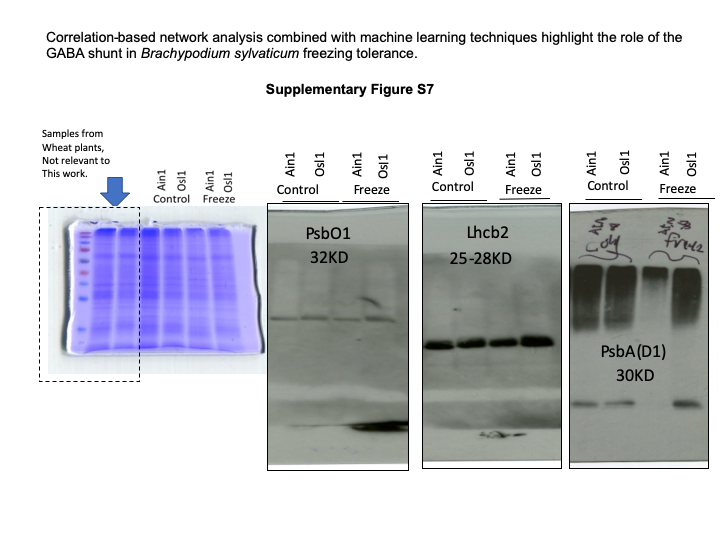

Supplement: Supplementary file 9 — Supplementary figure 7. [file 41598_2020_61081_MOESM9_ESM.tiff]
